# Neurological and Psychological Characteristics of Young Nitrous Oxide Abusers and Its Underlying Causes During the COVID-19 Lockdown

**DOI:** 10.3389/fpubh.2022.854977

**Published:** 2022-06-02

**Authors:** Gang Wu, Shanshan Wang, Tingling Wang, Jiali Han, Anna Yu, Changqiang Feng, Yajing Wang, Suzhi Liu

**Affiliations:** ^1^Department of Pharmacy, Taizhou Hospital of Zhejiang Province Affiliated to Wenzhou Medical University, Linhai, China; ^2^Department of Neurology, Taizhou Hospital of Zhejiang Province Affiliated to Wenzhou Medical University, Linhai, China; ^3^Department of Mental Health, Taizhou Hospital of Zhejiang Province Affiliated to Wenzhou Medical University, Linhai, China

**Keywords:** COVID-19, nitrous oxide, neurological, psychological, subacute combined degeneration of the spinal cord

## Abstract

**Background:**

The COVID-19 pandemic has a serious impact on the mental health of the public due to its economic and social impact. And psychological effects have led to drug and alcohol abuse. After the city lifted the lockdown, we consecutively encountered several young nitrous oxide abusers admitted to hospital for neurological treatment.

**Purpose:**

To inform physician decisions and social intervention, this observational study aimed at investigating the neurological and psychological characteristics of nitrous oxide abusers and its underlying causes during the COVID-19 lockdown.

**Methods:**

The nitrous oxide abusers who sought neurological treatment at our hospital between May 2020 and June 2020 were enrolled. Clinical data including socio-demographic, physical examination, laboratory examination, electromyography and neuroimaging were collected. Their motivations for inhaling nitrous oxide, knowledge about the nitrous oxide abuse and the accompanying of family were investigated face to face. Psychological status was assessed by the Symptom Checklist 90 (SCL-90) psychological evaluation.

**Results:**

Six nitrous oxide abusers were enrolled and the age was 22 ± 4.3. Clinical presentations included varying degrees of limb numbness and an ataxic gait. Laboratory examination revealed that all the patients did not have pernicious anemia, 4 patients had decreased vitamin B12 while 3 patients exhibited elevated homocysteine levels. MR of the spinal cord revealed that 4 patients had abnormal signals in the cervical spinal cord of high symmetry with splayed or inverted V sign after T2WI. Electromyogram (EMG) test showed 5 patients had peripheral nerve damage. The SCL-90 psychological evaluation results indicated that all patients had severe anxiety, depression and psychosis and they had severer psychological problems than ordinary citizens. Their motives for inhaling nitrous oxide are to relieve boredom, curiosity and buddy pressure. Their family spent <1 day per week to stay with them during city lockdown.

**Conclusion:**

The enrolled patients caused by abuse of nitrous oxide presented with symptoms of subacute combined with spinal degeneration. They had more serious psychological problems related to the COVID-19 pandemic. These cases make us value the psychological problems of young people under the outbreak and take multi-layered measures from families, schools (companies), hospitals, and governments to address it.

## Introduction

The COVID-19 pandemic and the ensuing lockdown have had a serious impact on the physical and mental health of the public (1, 2). And the psychological effects have led to drug and alcohol abuse (3). After the city lifted the lockdown, we consecutively encountered some young nitrous oxide abusers who were admitted to hospital for neurological treatment, which was a significant increase compared with the same period. It is important to characterize their neurological and psychological outcomes and explore the underlying causes in order to improve the clinical management during the COVID-19 pandemic period.

## Materials and Methods

### Study Design and Participants

Patients attending Taizhou Hospital of Zhejiang Province for care due to nitrous oxide abuse were consecutively enrolled. Clinical data including sociodemographic characteristics, physical examination, laboratory examination, electromyography, neuroimaging, and psychological assessment were obtained. The duration and frequency of nitrous oxide use, the sources of laughter were inquired. Their motivations for inhaling nitrous oxide were investigated face to face. The family environment, siblings, interpersonal relationships, personality traits, financial conditions, and academic performance were investigated. The survey about the time their parents or family member spent to stay with them and knowledge about the nitrous oxide abuse was carried out. The history of physical illness and family history were recorded. After the city was unsealed, the first two nitrous oxide abuser came to the hospital for neurological treatment at the same time. We thought it might be a phenomenon and therefore started this observational study. The study was done between May 2020 and June 2020.

All data were anonymized to comply with the provisions of personal data protection. The participants have provided their consent to publish the observational study, and the consent procedure was approved by the Ethics Committee of Taizhou Hospital of Zhejiang Province. All procedures were performed according to the guidelines of the institutional ethics committee and the tenets of the Declaration of Helsinki were adhered to throughout.

### Magnetic Resonance Imaging (MRI)

An MRI scan of the cervical spine and brain was done to all patients. T1WI sequences included MRI sequences with and without gadolinium. Sagittal and axial images were obtained using T2-weighted MRI sequences. Data on the affected spinal cord segments (number of segments of the spine) and their positions on the sagittal image (cervical and thoracic vertebrae) were recorded.

### Electromyogram (EMG)

Neurologic manifestations such as muscle weakness, sensory loss, and cognitive decline were recorded. Nerve conduction studies were performed on the median nerve, ulnar nerve, peroneal nerve, tibial nerve, and sural nerve depending on the clinical manifestations of patients. Compound muscle action potential (CMAP) amplitude, distal latency, sensory nerve action potential (SNAP) amplitude, and conduction velocity were detected using a full range functional EMG evoked potentiometer (Keypoint 9033A07, Denmark).

### Psychological Assessment

Using Symptom Checklist 90 (SCL-90), the mental state of the patients and ordinary citizens was assessed by a professional psychiatrist. The severity of symptoms (normal, mild, moderate, partial severe, severe, degree from light to heavy) is determined by the number of standard deviations of the dimension score from the norm group mean.

### Statistical Analyses

Data were analyzed by the Statistical Package for Social Sciences (IBM SPSS 16.0). Descriptive statistics and one-sample *t*-test were performed for data comparison between nitrous oxide abusers and ordinary citizens. Statistical significance was set at *P* ≤ 0.05.

## Results

### Sociodemographic and Laboratory Characteristics of the Patients

From May 2020 to June 2020, six patients with nerve damage caused by nitrous oxide inhalation were consecutively admitted to our hospital. As shown in Table 1, the mean age of the 6 patients was 22 ± 4.3, four were college students while two were high school graduates. The average duration of nitrous oxide abuse was 6.5 ± 4.4 months. Three of them began inhalation of nitrous oxide after 3^rd^, Feb, the day the city began lockdown. They consumed 240–720 nitrous oxide per time and 1–3 times per week. Nitrous oxide is bought in recreation place or through friends. Most of the patients have decreased vitamin B12 and increased homocysteine.

**Table 1:** Sociodemographic and laboratory characteristics of the case series.

**Case**	**Age**	**Sex**	**Education level**	**Duration of N2O Use (month)**	**Frequency of N2O Use**		**Vitamin B12** **level (pg/ml)**	**Homocysteine** **(μmol/L)**
					**Before 3rd, Feb (/time, times/month)**	**After 3rd, Feb** **(/time,** **times/week)**		
1	19	Male	College student	12	240–480, 4–5	240–480, 2–3	137.3	36.5
2	25	Female	High school	3	0	240–480, 1–2	262.7	7.9
3	28	Male	High school	3	0	240–480, 2–3	139.1	41.2
4	18	Female	College student	12	240–480, 4–6	480–720, 2–3	120.5	12^*^
5	22	Female	College student	3	0	480–720, 2–3	340.6^*^	10.5^*^
6	17	Female	College student	6	240–480, 3–4	240–480, 2–3	112.5	22

*N2O, nitrous oxide*.

* *Values after 2 days medicine treatment*.

Half of the patients were only children and half had one sibling. One had a poor interpersonal relationship with his family, and one was doting by his parents. All the patients did not live with their family and their parents or family members spent <2 h a day or 1 day per week to stay with or care for them during city lockdown. Their personality traits were either introverted, or withdrawn, or perverted. Five patients were in good economic condition and one was moderate. Five patients had moderate academic performance and one was lower. In addition, they didn't know that nitrous oxide abuse could lead abnormality of neurological function.

The patients stated that the reasons for nitrous oxide abuse were the lack of employment or study during the pandemic, a history of nitrous oxide abuse and relapse during the pandemic, boredom, curiosity and peer pressure.

### Neurological Characteristics of the Patients and Therapeutic Process

In the physical examine, all patients presented with limb numbness and varying degrees of walking instability. Varying degrees of sensory impairment and sensory ataxia were exhibited among the patients. There was no case of a positive pathological sign or obvious damage to the pyramidal tract. EMG examination showed peripheral nerve damage in patients except case 6. The abusers had multiple motor and sensory axonal damage and myelin sheath change, or motor nerve damage, or partial nerve damage. One case (case 4) had decreased muscle strength. Mild memory loss was documented in 2 patients (data not shown).

**Figure 1:**
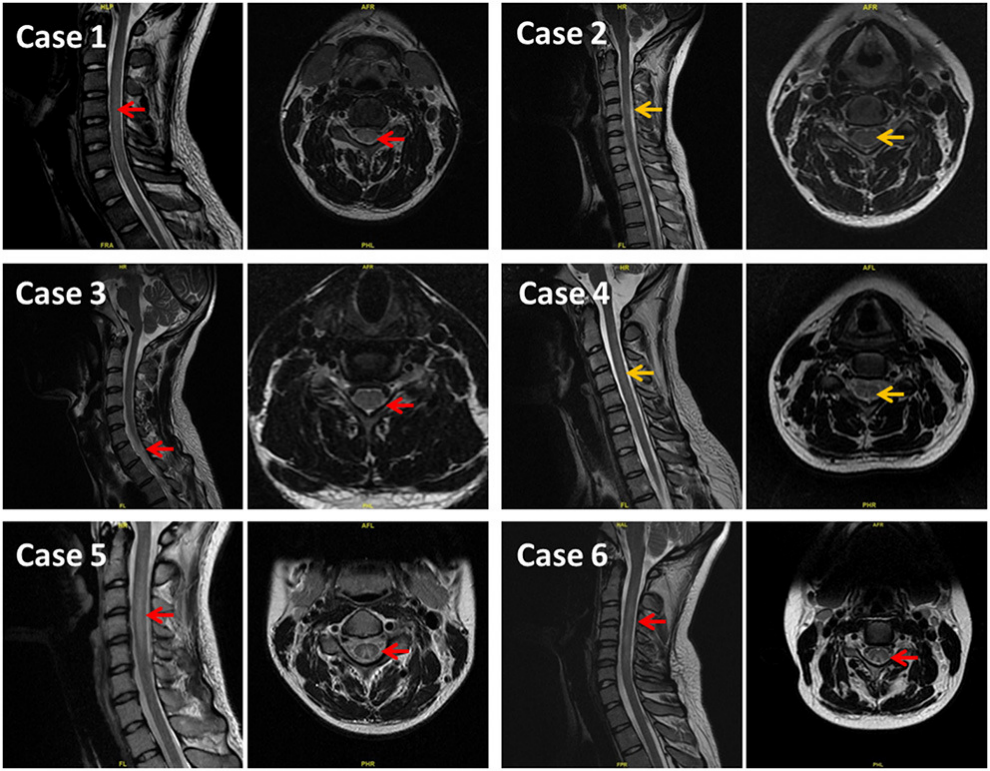
Results of MR T2WI showing high symmetry signal in the cervical spinal posterior cord, presenting splayed or inverted V sign. On the left of each case is sagittal image and on the right is axial image. Red arrows indicate a clearly high signal. Yellow arrows indicate slightly higher or suspicious signals.

The imaging results showed that four patients had high symmetric signals with splayed or inverted V signs in the cervical spinal cord after MR T2WI. Case 4 exhibited a slightly high signal while case 1 did not exhibit any imaging abnormalities (see Figure 1). All the patients presented no symptoms caused by autoimmune encephalitis, intracranial infection, cerebrovascular disease, brain trauma, tumor, or other toxic/metabolic causes, etc. All were diagnosed with subacute combined degeneration of the spinal cord (SCD). The six patients had improved neurological outcomes after vitamin B12 and adenosine cobalamin therapies for 5–8 days and discharged. They were prescribed vitamin B12 on discharge and told to return do a follow-up check on time.

All the patients had no history of physical illness or family history.

### Psychological Characteristics of the Nitrous Oxide Abusers

The SCL90 psychological evaluation results showed that the total score was 303.7 ± 43.1, each case was more than 250 points, and all cases had various psychological problems. The major severe psychological manifestations were anxiety, depression, hostility and psychosis (see Table 2).

**Table 2:** The results of psychological assessment by Symptom Checklist 90 evaluation.

**Case**	**Somatization**		**Obsessive-compulsive**		**Interpersonal sensitivity**	
	**Average score**	**Degree**	**Average score**	**Degree**	**Average score**	**Degree**
1	2.92	Partial severe	3.4	Partial severe	3.78	Partial severe
2	2.5	Moderate	2.4	Mild	4	Partial severe
3	4.33	Severe	3.7	Partial severe	4.11	Severe
4	3.5	Severe	2.2	Mild	2.67	Mild
5	3	Partial severe	3.3	Partial severe	4	Partial severe
6	2.25	Moderate	2.7	Moderate	3.33	Moderate
**Case**	**Depression**		**Anxiety**		**Hostility**	
	**Average score**	**Degree**	**Average score**	**Degree**	**Average score**	**Degree**
1	4.15	Severe	3.8	Severe	4	Severe
2	3.31	Partial severe	4	Severe	3.5	Partial severe
3	4.62	Severe	4.2	Severe	4	Severe
4	3.62	partial severe	3.4	severe	4	severe
5	4.08	Severe	3.7	Severe	3.5	Partial severe
6	3.08	Partial severe	2.9	Severe	3.83	Severe
**Case**	**Phobic anxiety**		**Paranoid ideation**		**Psychoticism**	
	**Average score**	**Degree**	**Average score**	**Degree**	**Average score**	**Degree**
1	3.57	Severe	3.33	Partial severe	3.43	Severe
2	2.71	Partial severe	3	Moderate	3.2	Severe
3	4.14	Severe	3.67	Severe	4.2	Severe
4	1.57	Normal	2.5	mild	3.2	severe
5	3.86	Severe	3	Moderate	3	Partial severe
6	2.71	Partial severe	4	Severe	2.3	Moderate

To analyze the differences in the psychological status of nitrous oxide abusers and normal people, the SCL-90 score of both groups was compared. The SCL-90 score of ordinary citizens under the level I emergency response was reported in a study which consisted sample size of 1,060 participants (1). It was noted that the SCL-90 scores of nitrous oxide abusers in anxiety, hostility, depression, interpersonal relationships, paranoia, psychosis and somatization were significantly higher than those of health controls, *P* < 0.01(Table 3), indicating that these young nitrous oxide abusers presented severer psychological problems than ordinary citizens of the same age. In view of this situation, all the patients were asked to go to the psychological department for check-up after discharge from hospital.

**Table 3:** Psychological status of the case series according to SCL-90, compared to ordinary citizens during COVID-19.

**Dimension**	**Case series** **(*n* = 6)**	**Ordinary citizens** **(*n* = 1,060)**	***t***	***P***
Somatization	3.08 ± 0.75	1.81 ± 0.69	4.171	0.009
Obsessive-compulsive	2.95 ± 0.6	2.24 ± 0.75	2.887	0.034
Interpersonal sensitivity	3.65 ± 0.55	2.06 ± 0.73	7.019	0.001
Depression	3.81 ± 0.58	1.96 ± 0.70	7.856	0.001
Anxiety	3.67 ± 0.46	1.91 ± 0.71	9.287	0.000
Hostility	3.81 ± 0.25	1.86 ± 0.68	19.426	0.000
Phobic anxiety	3.09 ± 0.95	2.03 ± 0.74	2.738	0.041
Paranoid ideation	3.25 ± 0.54	1.93 ± 0.71	6.04	0.002
Psychoticism	3.22 ± 0.62	1.88 ± 0.69	5.331	0.003

## Discussion

Due to the COVID-19 pandemic and the lockdown that followed, public psychological problems cannot be ignored. In addition to the heightened mental stresses among patients and healthcare workers during the COVID-19 pandemic, the mental health of healthy people was also affected. There was a drastic increase in public fear, a decline in social and economic activities that triggered psychosocial sequelae. Quarantined individuals exhibited depression, fear, guilt and anger (4). Psychosocial stress due to social changes in response to COVID-19 infections enhanced mental problems (1, 2, 5). In their study, Cuiyan Wang et al. reported that a total of 53.4% of the respondents exhibited either moderate or severe psychological problems under impact of the pandemic, 16.5% exhibited moderate to severe depressive symptoms, 28.8% had moderate to severe anxiety symptoms while 8.1% had moderate to severe stress (6). The psychological effects lead to drug and alcohol abuse (3, 7). And it is notable that these problems are more likely to happen among children and adolescents (8–10).

After the city lifted the lockdown, we consecutively encountered 6 nitrous oxide abusers who were hospitalized for neurological therapy within 1 month and they were all youth. It is notable that there were only 6 patients of nitrous oxide abuse were treated between October 2017 and December 2019 in our hospital and it cued the effect of the COVID-19 pandemic and the lockdown on public health especially the young. For physician decisions and social intervention, it was urgent to investigate the neurological characteristics and psychological state of them and the underlying causes of nitrous oxide abuse during the COVID-19 lockdown.

For more than 170 years, nitrous oxide has been used as an anesthetic in clinical practice. Its inhalation causes feelings of euphoria, involuntary laughter, distorted voices and mild hallucinations and it gradually becomes a popular way to relieve the pressures among the youth (11, 12). A global drug survey (GDS2014) conducted in 17 countries involving 74,864 patients confirmed that the prevalence of nitrous oxide use as a recreational drug in the UK and US was 38 and 29.4%, respectively (12). Incidences of nitrous oxide abuse in China are gradually increasing, with the majority of the abusers being the youth (11).

The adverse effects of exposure to nitrous oxide include slowed reaction rate, dizziness, nausea and vomiting. Inhalation of large quantities of nitrous oxide at a high pressure may lead to suffocation. Long-term adverse effects include nerve damage due to vitamin B12 deficiency, cobalamin reactive psychosis, and homocysteine accumulation (13). Vitamin B12 is an important cofactor of cellular methionine synthase. Extremely low levels of vitamin B12 leads to methionine consumption and homocysteine accumulation. Methionine consumption leads to a decrease in downstream S-adenosine, which is required for myelin production and maintenance. Deficiency in vitamin B12 leads to demyelination and gliosis of the central nervous system (especially the dorsal spinal cord), as well as demyelination of peripheral nerves. Homocysteine accumulation increases the risk of stroke and peripheral neuropathy (14).

Pernicious anemia and neurological damage caused by nitrous oxide are very common. Clinical manifestations of these conditions include paresthesia in limbs, gait instability or difficult walking, weakness, falls or balance disorders, Lhermitte's Sign and ataxia (15). Occasionally there is cognitive impairment and optic atrophy (14, 16). In this study, all the 6 nitrous oxide abusers presented with limb numbness and varying degrees of walking instability. Two patients presented with mild memory loss, 4 presented with increased T2 signal in cervical spinal cord, 3 presented with extensive peripheral nerve damage, while 1 exhibited mild anemia. In terms of treatment, the neurological symptoms could be ameliorated by in time vitamin B12 supplementary (13, 17). All the patients had improved neurological outcomes after vitamin B12 therapies and discharged.

Considering the impact of the COVID-19 epidemic on people's mental health (18), the psychological assessment was carried out. The results indicated varying degrees of anxiety, depression, hostility and psychosis and one case presented obvious suicidal tendency. The SCL-90 score of the 6 patients was significantly higher compared to that of healthy individuals. During COVID-19 pandemic and city lockdown, stressors such as university closures, social distancing, pessimism on the economic prospects are susceptible to lead development of mental health symptoms (18). Compared with the past, the increase in the number of hospitalizations caused by nitrous oxide abuse, and the increase of nitrous oxide dose or relapse reflected to a certain extent the impact of the COVID-19 pandemic on people's psychological status.

To explore the potential causes of nitrous oxide abuse during the COVID-19 lockdown, motivations for inhaling nitrous oxide, sociodemographic characteristics, family environment, siblings, interpersonal relationships, personality traits, financial conditions, and academic performance were investigated. The results showed that risk factors for nitrous oxide abuse included the lack of employment or study during the pandemic, a history of nitrous oxide abuse and relapse during the pandemic, boredom, curiosity and peer pressure, parental or family inconcern or doting, and possible good economic situation.

Although there were still many debates about the lockdown policy (19), it did inhibit the spread of the SARS-CoV-2 and reduce the absolute number of deaths (20). We should focus more on solving the problems caused by the city lockdown such as the psychological problems and take effective measures for the above potential causes. It should enhance the combination of meaning-based coping and spirituality processes to mitigate the adverse effects of coronavirus stress on wellbeing (21). Multi-layered interventional measures from families, schools, hospitals, and governments should be implemented as early as possible. It is worth emphasizing that the patients' family rarely communicated with them during the lockdown. Loneliness is strongly associated with mental health problems (22). Therefore, the company of the family appears to be extremely important (23). Indoor games, read and physical sports with the family are recommended. Despite of social distancing and school closures, on-line courses and virtual workshops where clinician-led mental health and psychosocial services such as stress control, drug abuse education are conducted should be encouraged. For those with obvious suicidal tendency, severe depression or other serious psychological problems, drug therapy intervention by psychiatrist needs to be involved (24). Lastly, government's measures should be taken to control the nitrous oxide flooding from the source such as recreation places (11).

Disadvantage of this study: nitrous oxide abuse not only leads to peripheral neuropathy, SCD and other physiological diseases (25–27) but also causes a series of abnormal mental symptoms, including personality changes, mood disorientations (such as anxiety, depression, mania), impulsive and aggressive behaviors, hallucinations, delusions and other psychotic symptoms (28). We failed to obtain the psychological assessment data of the patients before the pandemic and before they started abusing nitrous oxide. The causal relationships between the pandemic and psychological changes, and between nitrous oxide abuse and psychological changes could not be explained. The second disadvantage is that the psychological status of the general population in the city was not obtained at the time of psychological assessment of the patients.

## Conclusion

The nitrous oxide abusers during the COVID-19 pandemic and lockdown presented SCD neurological symptoms and more serious psychological problems than healthy controls. In addition to the neurological therapy, more attention should be payed to the mental health of them. These young cases make us value the psychological problems of young people under the outbreak and it is imperative to take multi-layered, three-dimensional measures from families, schools (companies), hospitals, and governments to address it.

## Data Availability Statement

The raw data supporting the conclusions of this article will be made available by the authors, without undue reservation.

## Ethics Statement

The studies involving human participants were reviewed and approved by Ethics Committee of Taizhou Hospital of Zhejiang Province. Written informed consent to participate in this study was provided by the participants' legal guardian/next of kin.

## Author Contributions

GW, SW, and SL were involved in conception and design, case diagnosis, data analysis, data interpretation, critical review, and manuscript drafting for this article. TW, CF, AY, YW, and JH were involved in data collection. GW and JH contributed to manuscript revision. All authors reviewed and confirmed for this article. All authors contributed to the article and approved the submitted version.

## Funding

This work was supported in part by grants from National Natural Science Foundations of China (No. 81903584 to GW); Zhejiang Provincial Basic and Public Welfare Research Program (LGD20H310002 to GW and LGF20H090009 to SL).

## Conflict of Interest

The authors declare that the research was conducted in the absence of any commercial or financial relationships that could be construed as a potential conflict of interest.

## Publisher's Note

All claims expressed in this article are solely those of the authors and do not necessarily represent those of their affiliated organizations, or those of the publisher, the editors and the reviewers. Any product that may be evaluated in this article, or claim that may be made by its manufacturer, is not guaranteed or endorsed by the publisher.

## Abbreviations

SCL-90: Symptom Checklist 90
EMG: Electromyogram
MRI: Magnetic Resonance Imaging
CMAP: Compound muscle action potential
SNAP: Sensory nerve action potential
SCD: Subacute combined degeneration of the spinal cord
COVID-19: Corona Virus Disease 2019
SARS-CoV-2: Severe Acute Respiratory Syndrome Coronavirus 2.
